# Patient and Staff Safety Incidents in Korean Dental Practice: Implications for Quality of Care and Safer Healthcare Delivery

**DOI:** 10.3390/healthcare14131895

**Published:** 2026-06-30

**Authors:** Kyeol Koh, Se Hoon Kahm

**Affiliations:** 1 Department of Dentistry, Eunpyeong St. Mary’s Hospital, College of Medicine, The Catholic University of Korea, Seoul 03312, Republic of Korea; kohkyeol@catholic.ac.kr; 2 Department of Dentistry, Graduate School of Chosun University, Gwangju 61452, Republic of Korea; 3 College of Medicine, The Catholic University of Korea, Seoul 06591, Republic of Korea; 4 Dental Implantology, Graduate School of Clinical Dental Science, The Catholic University of Korea, Seoul 06591, Republic of Korea

**Keywords:** patient safety, quality of care, workplace violence, occupational safety, healthcare delivery

## Abstract

**Highlights:**

**What are the main findings?**

- In 439 Korean dental professionals, 89.1% reported at least one lifetime safety incident.
- The most common events were foreign body aspiration/ingestion, instrument-related patient injury, sharps injury, and verbal abuse.

**What are the implications of the main findings?**

- Patient safety and occupational safety should be integrated into quality-improvement efforts in dental healthcare.
- Targeted education and institutional safety systems may help reduce risk and support safer care delivery.

**Abstract:**

**Objectives**: Patient safety is central to healthcare quality, yet dental practice also involves occupational risks for professionals. This study examined the lifetime prevalence and types of patient- and staff-safety incidents among Korean dental professionals and explored associated demographic, professional, and institutional factors. **Methods**: A cross-sectional survey was conducted among 439 dental professionals in South Korea. Participants reported lifetime experience of predefined safety incidents, institutional safety factors, and demographic and occupational characteristics. Descriptive statistics, profession-based comparisons, and multivariable logistic regression were applied. **Results**: Overall, 89.1% of respondents reported at least one safety incident. The most common patient-safety events were aspiration or ingestion of teeth or prosthetic materials and instrument-related injury, whereas sharps injuries and verbal abuse were the leading staff-safety issues. Dentists and dental hygienists differed significantly in response knowledge, liability insurance coverage, and safety education. The presence of institutional safety protocols was associated with higher reported incident experience, which may reflect greater recognition and reporting rather than a causal increase in harm. **Conclusions**: Safety incidents are highly prevalent in Korean dental practice and represent an underrecognized quality-of-care and workforce-safety issue. Integrated strategies including occupational-hygiene measures, structured safety education, non-punitive reporting, and stronger organizational preparedness are needed to improve dental healthcare delivery.

## 1. Introduction

Patient safety has become a global priority in healthcare, focusing on the prevention or reduction of avoidable harm occurring during medical services [1,2,3]. Patient safety concerns are increasingly relevant in dental environments, where distinctive risks, including foreign body ingestion or aspiration, chemical injury, wrong-tooth procedures, instrument-related injury, and nerve damage, may occur during routine care [4,5]. Dental care is also delivered through multidisciplinary teams involving dentists, dental hygienists, assistants, nurses, students, and administrative staff, and therefore patient safety and workforce safety are closely linked within the same service system. Evolving technologies, including digital and AI-assisted systems, may introduce new complexities in communication, documentation, workflow design, and safety management [6,7]. Despite these considerations, dental safety research has often examined patient adverse events and occupational hazards separately, limiting a more integrated understanding of healthcare quality in dental practice.

An important but comparatively underexplored dimension is staff safety. Dental professionals frequently encounter occupational hazards, including sharps injuries, bio-aerosol exposure, chemical handling, musculoskeletal strain, and patient-initiated violence such as verbal abuse and physical assault [8,9,10,11,12,13]. In Korea, dentists are licensed to diagnose and provide dental treatment, whereas dental hygienists are licensed healthcare personnel whose legal and practical duties focus on oral disease prevention, dental hygiene management, radiography, impressions, temporary procedures, and treatment assistance under the dental care system [14]. These differences in scope of practice, clinical authority, and procedure-related exposure may create different safety-risk profiles. Although several Korean studies have examined patient adverse events or workplace violence separately, evidence that jointly characterizes patient- and staff-safety incidents in a single sample of dental professionals is limited. This study aimed to investigate the lifetime prevalence and distribution of patient- and staff-safety incidents among Korean dental professionals and to identify demographic and institutional factors associated with incident experience, with implications for quality improvement, workforce protection, and safer healthcare delivery in dental settings.

## 2. Materials and Methods

### 2.1. Ethical Approval

This study was conducted in accordance with the World Medical Association Declaration of Helsinki and was approved by the Institutional Review Board (IRB) of The Catholic University of Korea, Catholic Medical Center (approval number PC23QISI0037, 16 March 2023). Because the survey was anonymous, voluntary, and posed no more than minimal risk to participants, the IRB waived the requirement for written documentation of informed consent; an information sheet describing the study purpose, voluntary nature, anonymity, and data use was provided to all participants prior to participation, and completion of the questionnaire was treated as implied consent.

### 2.2. Study Design and Participants

**Figure 1:**
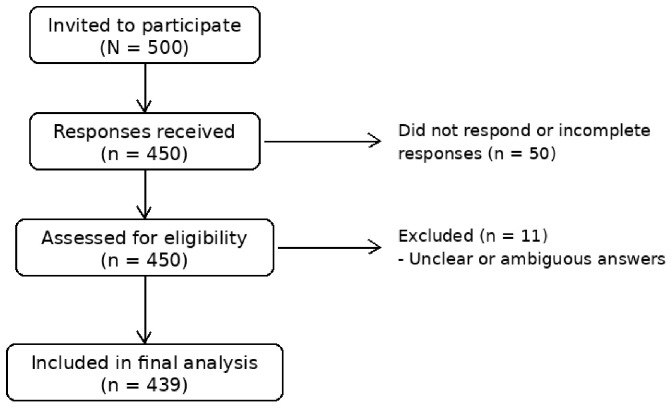
Flow diagram of participant selection.

A descriptive, cross-sectional survey targeting approximately 500 dental professionals across South Korea yielded 450 responses after initial screening; 11 were subsequently excluded for ambiguous answers, giving a final sample of 439 valid responses (Figure 1). Respondents were asked to report their lifetime (career-to-date) experience of each pre-defined safety incident type; unless otherwise specified, all reported prevalence figures refer to this lifetime recall period.

### 2.3. Data Collection Instrument

The survey instrument was developed from prior Korean and international literature on dental adverse events, medical disputes, infection prevention, occupational hazards, and workplace violence [4,5,8,9,10,11,12,13,15,16,17,18,19]. It comprised four sections: (1) demographic and occupational characteristics; (2) incident experience status (dichotomous yes/no); (3) incident types and frequency (18 predefined incidents across patient-safety and staff-safety categories); and (4) safety management factors (protocol knowledge, training experience, liability insurance status, and institutional protocol presence). The predefined categories were intended to cover clinically recognizable dental patient-safety events and occupational-safety domains, including foreign body aspiration/ingestion, instrument injury, chemical injury, systemic emergencies, sharps injury, biting, and workplace violence.

Institutional safety protocol status was assessed using a single self-reported item asking whether the respondent’s institution had an established safety protocol or response guideline. This variable should therefore be interpreted as the presence of a protocol rather than the robustness of emergency preparedness. The survey did not collect detailed information on protocol contents, such as regular accident-response drills, tiered response manuals, designated reporting officers, written standard operating procedures for aerosols, chemical spills or sharps disposal, or post-incident root-cause analysis.

The questionnaire was reviewed for content relevance and clinical clarity by a small expert panel consisting of dental clinicians and dental-hygiene/quality-management professionals with experience in dental clinical practice, patient safety, or dental education. The expert review focused on whether the incident categories were understandable to Korean dental professionals and whether they reflected common dental safety concerns. The categories were conceptually informed by international patient-safety and infection-prevention guidance, but the instrument was not designed as a formal psychometric scale. No content validity index, construct validation, internal consistency assessment, or test–retest reliability assessment was performed; this is acknowledged as a limitation. The panel comprised five reviewers, including dentists with hospital-based safety-and-quality experience, a dental hygienist with infection-control responsibilities, and a healthcare-quality professional, so that clinical, occupational-hygiene, and methodological perspectives were represented (see details in the Supplementary Materials).

### 2.4. Statistical Analysis

Data were analyzed using SPSS 25.0 (IBM Corp., Armonk, NY, USA). Descriptive statistics and chi-square or Fisher’s exact tests were applied where appropriate. Profession-based comparisons were used to examine differences between dentists and dental hygienists, and demographic variables such as gender, age, career duration, and education level were summarized to support risk-stratified interpretation. Two multivariable binary logistic regression models were constructed: Model 1 (overall safety incident experience) and Model 2 (staff safety issue experience), with gender, age, profession, career duration, education level, protocol presence, and frequency of treating patients with disabilities as independent variables. Results are expressed as odds ratios (OR) with 95% confidence intervals (CI); *p* < 0.05 was considered statistically significant.

During the preparation of this work, the authors used ChatGPT 5.2 (OpenAI, San Francisco, CA, USA) to assist with English-language editing and translation of parts of the manuscript and questionnaire. After using this tool, the authors reviewed and edited the content as needed and take full responsibility for the content of the published article.

## 3. Results

### 3.1. General Characteristics and Safety Incident Experience

A total of 439 dental professionals participated. Females (70.8%, n = 311) outnumbered males (29.2%, n = 128). Dental hygienists comprised 56.3% (n = 247), dentists 41.7% (n = 183), and a small group of other dental staff (e.g., nurses and assistants working in dental settings) accounted for the remaining 2.0% (n = 9). The overall incident experience rate was 89.1% (n = 391/439). Stratified descriptively, the rate was similar by gender (90.6% in males and 88.8% in females) and age group (87.1% under 30 years, 89.6% at 30–39 years, and 89.3% at 40 years and older). By profession, 92.9% of dentists and 88.3% of dental hygienists reported at least one incident, while the highest descriptive rate by career duration was observed in the 6–10 years group (93.5%) (Table 1).

### 3.2. Frequency and Proportion of Safety Incident Types

A total of 2489 safety incidents were reported. The most frequent was ‘aspiration or ingestion of teeth or prosthetics’ (64.0%, n = 281), followed by ‘patient injury by dental instrument’ (62.2%, n = 273). Staff-related hazards were also prominent: sharps injuries were reported by 233 respondents (53.1%), and chemical-induced patient skin or clothing damage was reported by 162 respondents (36.9%). Workplace violence was reported by 198 respondents (45.1%), with verbal abuse being the most prevalent subtype (39.4%, n = 173) (Table 2).

### 3.3. Comparison of Safety Factors by Profession

Statistically significant disparities were observed between dentists and dental hygienists across all safety management factors (*p* < 0.001 for all): accident response knowledge (78.69% vs. 57.49%), liability insurance coverage (65.57% vs. 26.32%), and safety education experience (73.77% vs. 44.94%) (Table 3).

### 3.4. Factors Associated with Overall Safety Incident Experience

Multivariable logistic regression identified education level, protocol status, and frequency of treating patients with disabilities as significant predictors of overall safety incident experience (Table 4). The presence of a safety protocol was significantly associated with increased likelihood of reported incidents (OR = 2.122, *p* = 0.036). Because the protocol variable captured only the reported presence of a protocol and not its maturity or implementation quality, this result should be interpreted cautiously and may reflect greater incident recognition, reporting, or post-incident protocol adoption rather than a harmful effect of protocols themselves. Descriptive cross-tabulations of incident experience by sex and age band (see Table 1) suggested broadly similar rates across groups, with the highest rate in the 6–10-year career group (93.5%); these patterns are consistent with cumulative lifetime exposure increasing with career duration and are interpreted further in the Discussion.

### 3.5. Analysis of Staff Safety Issues

Sharps injury (53.1%) and verbal abuse (39.4%) were the most frequently reported staff safety issues, with significant profession-based differences across staff safety categories (Table 5). In the multivariable model, gender (female), profession (dental hygienist), highest education (Ph.D. or higher), and protocol status were significant predictors of staff safety issue experience (Table 6). These findings suggest that demographic and professional characteristics may influence reported staff-safety risk, although interpretation should consider the composite nature of the outcome.

## 4. Discussion

### 4.1. Safety Incidents, Reporting Behavior, and Implications for Healthcare Quality

Our findings indicate that safety incidents in dental settings should be understood not only as isolated clinical mishaps but also as indicators of broader quality-of-care challenges within healthcare delivery. Although direct comparison is difficult because of differences in definitions, measurement periods, and incident scope, the observed lifetime rate appears high relative to figures reported in some previous studies [15,16,17]. A U.S. study reported 45.5% physical and 74.0% verbal aggression over an entire career [15,16], while a Korean survey found that 70% of dentists experienced patient violence or threats [17]. International dental incident-reporting data also show that patient, personnel, and organizational safety incidents coexist within dental hospitals, while under-reporting can substantially limit interpretation of the true frequency and pattern of adverse events [20]. These differences reinforce the need to interpret prevalence estimates in relation to study design, local reporting culture, and the distinction between self-reported lifetime experience and formally reported incidents.

A counter-intuitive finding emerged: the presence of a safety protocol was associated with a higher likelihood of both overall incident experience (OR = 2.122, *p* = 0.036) and staff safety issues (OR = 1.925, *p* = 0.016), an association we interpret cautiously in light of what has been termed the reporting paradox [21,22,23]. One plausible explanation is that institutions with protocols actively educate staff to recognize and report incidents, potentially increasing reported rates relative to settings without formal protocols; as observed in hospital safety research, stronger safety cultures can correlate with higher reporting, not necessarily higher actual harm [23,24]. However, given the cross-sectional design and the single-item assessment of protocol presence, this interpretation cannot be considered established. Reverse causation is also plausible: institutions that have already experienced incidents may be more likely to subsequently implement protocols. Unmeasured confounding by practice setting, such as academic and hospital-based clinics tending both to have more formal protocols and to handle a more diverse case mix, could also independently raise both protocol presence and incident exposure. Regardless of mechanism, the finding underscores the importance of non-punitive just culture, transparent reporting pathways, feedback to frontline staff, and root-cause analysis that leads to system redesign rather than blame [23,24].

### 4.2. Staff Safety: Occupational Hazards and Workplace Violence

These findings highlight workforce safety as an important component of healthcare quality in dental settings. Sharps injuries, chemical exposure, and bio-aerosol exposure require an occupational-hygiene approach rather than reliance on individual vigilance alone. Dental procedures can generate contaminated aerosols and splatter, and infection-prevention guidance recommends layered controls, including standard precautions, hand hygiene, appropriate PPE, instrument processing, environmental cleaning, and administrative procedures [9,10,11,12]. For high-risk procedures, institutions should consider the hierarchy of controls: elimination or substitution when feasible; engineering controls such as high-volume evacuation, rubber dam isolation, physical barriers, ventilation, closed sharps containers, and safer devices; administrative controls such as written standard operating procedures, training, pre-procedure planning, incident reporting, and vaccination/post-exposure pathways; and PPE such as masks or respirators, eye protection, face shields, gowns, and gloves. For chemical handling, protocols should include labeling, storage, spill management, eye and skin protection, and immediate response procedures. For sharps, prevention should include safe passing, avoidance of two-handed recapping, immediate disposal, post-exposure assessment, and reporting, because under-reporting of needlestick and sharps injuries is well documented in dental education and healthcare settings [13].

Workplace violence also constituted a substantial occupational burden. Multi-victimization was evident: the cumulative abuse count (n = 203) exceeded unique victims (n = 198), indicating overlapping forms of aggression. Previous international studies have reported similar patterns [25,26]: 70% of Saudi dental students experienced patient shouting [25], and 75% of female dentists experienced patient or guardian violence [26]. Multivariable analysis revealed that dental hygienists were less likely than dentists to experience staff safety problems (OR = 0.571, *p* = 0.038), and female workers less likely than males (OR = 0.520, *p* = 0.021). This may reflect differences in professional roles and exposure: dentists perform more invasive procedures and hold positions of direct clinical authority, increasing exposure to both procedural hazards and patient-initiated conflict. It is also important to note that the composite staff-safety outcome was driven primarily by sharps injury (n = 233), which dentists may experience more frequently by virtue of performing more invasive procedures, whereas violence-specific outcomes may show different patterns. Potential countermeasures include de-escalation training, clearer reporting pathways, organizational support, and, where appropriate, environmental security measures [17,27,28].

### 4.3. Disparities in Safety Competency and the Need for Targeted Education

Dentists reported higher levels of response knowledge, liability insurance coverage, and safety education than dental hygienists (response knowledge: 78.69% vs. 57.49%; liability insurance coverage: 65.57% vs. 26.32%; safety education: 73.77% vs. 44.94%; all *p* < 0.001), suggesting disparities in training opportunities and access to institutional support and resources. In Korea, dental hygienists are licensed healthcare personnel whose duties are centered on oral disease prevention and dental hygiene management, while their practical work may also include chairside assistance and radiography within the dental care system [14]. Because dental hygienists comprised the largest professional group in this sample and are often positioned at the interface between patients, instruments, infection-control procedures, and treatment support, lower access to safety education and liability protection is a quality-of-care concern rather than only an individual occupational issue.

A Korean study found that although 85% of dental hygienists recognized the importance of safety, only 35% reported direct incident experience, suggesting that safety awareness does not necessarily translate into incident reporting [29]. Dental students also face early exposure: Rhoades et al. demonstrated frequent patient aggression during clinical training [30], and Looper and Esfandiari reported that over half experienced aggression within one year [31]. These findings support structured safety curricula across dental and dental-hygiene education, simulation-based training for aspiration/ingestion and medical emergencies, sharps and chemical safety modules, violence de-escalation training, and continuing education tied to institutional incident-reporting systems [13,23,32]. Professional organizations and employers may also consider broader access to professional liability insurance and post-incident support for all dental team members, not only dentists.

### 4.4. Medical Disputes, Legal Frameworks, and Protective Factors

Korean studies have reported that medical accidents and related disputes are common in dental practice, with implant complications and foreign body ingestion identified as frequent triggers [18,19]. Case reviews and precedent analyses have consistently highlighted inadequate informed consent, poor record-keeping, and communication failures as major legal risk factors in dentistry [32,33,34]. The 43.9% rate of uncertainty regarding insurance status (n = 193) in the present study suggests that many practitioners may remain insufficiently prepared for the financial and legal consequences of adverse events. In addition, a Ph.D. or higher was associated with lower odds of staff safety issue experience (OR = 0.458), which may reflect reduced clinical exposure, seniority-related differences in task allocation, or greater familiarity with risk management.

At the policy level, Korea operates the Korea Patient Safety Reporting & Learning System (KOPS), a national information collection and learning system for patient-safety incidents operated on the basis of the Patient Safety Act [35]. However, the present survey could not be directly triangulated with KOPS or other external reporting datasets because the study relied on anonymous self-report and detailed publicly accessible dental-practice-specific reporting data were not available at the participant level. Future dental safety systems should aim to link confidential reporting, standardized classification, feedback to clinicians, education for students and staff, and organizational learning. For educators, this implies embedding safety science into curricula; for regulators, it suggests encouraging minimum safety-management expectations and reporting culture; and for employers, it supports regular drills, clear escalation pathways, safety equipment, and post-incident support.

### 4.5. Limitations

This study has several limitations. The cross-sectional design and self-reported data are susceptible to recall and social desirability bias; notably, the latter may operate bidirectionally, with possible under-reporting of stigmatized exposures such as workplace violence and possible over-reporting of socially desirable attributes such as safety protocol awareness. Convenience sampling raises the possibility of selection bias toward safety-conscious respondents, which may partly account for the unusually high 89.1% lifetime prevalence; this near-ceiling outcome also limits variance for binary logistic regression and may inflate odds ratios, so the magnitude of the reported associations should be interpreted with caution. The dataset does not include a detailed practice-setting variable (e.g., private dental office vs. dental hospital vs. university-affiliated clinic), which is a plausible confounder of the association between protocol presence and reported incidents and could not be adjusted for in the present models. Although demographic variables were included in descriptive and regression analyses, the available dataset did not support a full matrix of sex-by-age-by-profession cross-tabulations for each incident subtype without creating sparse cells, especially for less common events such as physical abuse and systemic emergencies. The protocol variable captured only the reported presence of a protocol and did not measure protocol quality, content, drill frequency, reporting infrastructure, or root-cause-analysis processes. The instrument was developed from prior literature and refined through expert content review only, without formal psychometric testing. Predefined incident categories may not capture the full safety spectrum, and the absence of detailed clinical incident data precludes verification of self-reports against records or national reporting systems such as KOPS. Logistic regression identifies associations, not causation, and findings are limited to South Korea and to survey-engaged practitioners. Future research should employ prospective designs, objective reporting systems, international samples, formally validated instruments, detailed practice-setting variables, and qualitative methodologies.

## 5. Conclusions

This survey of 439 Korean dental professionals indicates that patient and staff safety incidents are common, with nearly 90% reporting at least one lifetime event. Foreign body aspiration or ingestion, instrument injuries, sharps exposures, chemical-related injuries, and workplace violence represent major safety challenges. Systemic disparities in safety knowledge, training, and insurance between dentists and dental hygienists demand targeted interventions. Improving safety in dental settings requires integrated strategies that combine patient safety education, occupational-hygiene controls, PPE and sharps-management systems, workforce protection, organizational preparedness, non-punitive reporting, and feedback-driven quality improvement. Because dental safety concerns overlap with broader healthcare delivery issues, these findings may be relevant not only to dental professionals but also to educators, employers, regulators, and healthcare service providers seeking safer and more resilient clinical systems. Because the issues identified here—sharps and bio-aerosol exposure, chemical handling, workplace violence, and uneven institutional preparedness—are common to many healthcare service providers beyond dentistry, we hope the findings will support cross-disciplinary efforts toward safer, more equitable, and more resilient healthcare delivery.

## Figures and Tables

**Table 1:** General Characteristics of Participants and Safety Incident Experience (N = 439, 95% CI).

Category	Classification	N	Percentage (%)	Incident Experience Rate (%)
Gender	Male	128	29.16	90.63
	Female	311	70.84	88.75
Age	Under 30	70	15.95	87.14
	30–39	183	41.69	89.62
	40 and over	186	42.35	89.25
Profession	Dentist	183	41.69	92.90
	Hygienist	247	56.26	88.26
	Others	9	2.05	100.00
Career	1–5 years	91	20.73	86.81
	6–10 years	123	28.02	93.50
	11+ years	225	51.25	88.00
Total		439	100.00	89.07

**Table 2:** Frequency and Proportion of Patient Safety Incident Types by Profession (Top 10).

Rank	Accident Type	Dentist (N = 183)	Hygienist (N = 247)	Others (N = 9)	Total Count	Total Proportion (%)
1	Aspiration/Ingestion of teeth/prosthetics	101 (55.2%)	173 (70.0%)	7 (77.8%)	281	15.93
2	Patient Injury by Dental Instrument (puncture, cut, etc.)	130 (71.0%)	139 (56.3%)	4 (44.4%)	273	15.48
3	Sharps injuries among staff	114 (62.3%)	117 (47.4%)	2 (22.2%)	233	13.21
4	Verbal/Physical Abuse	99 (54.1%)	97 (39.3%)	2 (22.2%)	198	11.23
5	Patient Skin/Clothing Damage by Chemicals	80 (43.7%)	80 (32.4%)	2 (22.2%)	162	9.18
6	Sensory Nerve Damage after Dental Treatment	61 (33.3%)	95 (38.5%)	5 (55.6%)	161	9.13
7	Patient Allergy or Hypersensitivity Reaction	69 (37.7%)	64 (25.9%)	4 (44.4%)	137	7.77
8	Tooth Chart Error (treating the wrong tooth)	45 (24.6%)	76 (30.8%)	4 (44.4%)	125	7.08
9	Systemic Emergency (Hyperventilation, Syncope, Cardiac Arrest)	48 (26.2%)	58 (23.5%)	8 (88.9%)	114	6.46
10	Patient Injury by Cut (laceration, abrasion)	41 (22.4%)	51 (20.6%)	2 (22.2%)	94	5.33

**Table 3:** Comparison of Safety and Response Factors by Profession (N = 439; inferential comparisons exclude Others, n = 9; Bonferroni-adjusted alpha = 0.017).

Category	Classification	Dentist (N = 183)	Hygienist (N = 247)	Others (N = 9)	*p*-Value
Response Knowledge	Aware	144 (78.69%)	142 (57.49%)	9 (100.00%)	<0.001
Liability Insurance	Covered	120 (65.57%)	65 (26.32%)	5 (55.56%)	<0.001
Safety Education	Experienced	135 (73.77%)	111 (44.94%)	6 (66.67%)	<0.001

**Table 4:** Factors Associated with Overall Safety Incident Experience (Logistic Regression Analysis).

Independent Variable	Classification	B (SE)	OR (95% CI)	*p*-Value
Highest Education	Associate’s Degree (Reference)	-	1.000	-
	Bachelor’s Degree	0.005 (0.395)	1.005 (0.468–2.155)	0.990
	Master’s Degree	0.040 (0.443)	1.041 (0.438–2.470)	0.928
	Ph.D. or higher	−0.900 (0.435)	0.407 (0.173–0.959)	0.039 *
Protocol Status	No Protocol (Reference)	-	1.000	-
	Has Protocol	0.752 (0.355)	2.122 (1.050–4.288)	0.036 *
Disability Patient Treatment	Never Treat (Reference)	-	1.000	-
	Rarely Treat	−0.770 (0.364)	0.463 (0.229–0.938)	0.033 *
	Usually Treat	−0.428 (0.347)	0.652 (0.330–1.287)	0.219

* Note: B, unstandardized regression coefficient; SE, standard error; OR, odds ratio; CI, confidence interval. Dependent Variable: Safety Incident Experience (Yes = 1, No = 0). Significant at *p* < 0.05.

**Table 5:** Frequency of Staff Safety Issues by Profession (N = 439; inferential comparisons exclude Others, n = 9; Bonferroni-adjusted alpha = 0.013).

Staff Safety Issues	Dentist (N = 183)	Hygienist (N = 247)	Others (N = 9)	Total Count	Total Respondents (%)	*p*-Value
Sharps injuries among staff	114 (62.3%)	117 (47.4%)	2 (22.2%)	233	53.08%	<0.001
Verbal Abuse	99 (54.1%)	74 (29.9%)	0 (0.0%)	173	39.41%	<0.001
Biting by Patient	63 (34.4%)	65 (26.3%)	1 (11.1%)	129	29.38%	0.046 *
Physical Abuse	18 (9.8%)	12 (4.9%)	0 (0.0%)	30	6.83%	<0.001

* Note: The total count of violence incidents (verbal abuse n = 173 plus physical abuse n = 30; total event count n = 203) exceeds the number of unique victims (n = 198) reported in Table 2, as some respondents reported experiencing both verbal and physical abuse. Inferential comparisons exclude the ‘Others’ group (n = 9).

**Table 6:** Factors Influencing Staff Safety Issue Experience (Logistic Regression Analysis).

Independent Variable	Classification	B (SE)	OR (95% CI)	*p*-Value
Gender	Male (Reference)	-	1.000	-
	Female	−0.654 (0.285)	0.520 (0.298–0.908)	0.021 *
Profession	Dentist (Reference)	-	1.000	-
	Hygienist	−0.560 (0.270)	0.571 (0.337–0.967)	0.038 *
Highest Education	Associate’s degree (Reference)	-	1.000	-
	Bachelor’s Degree	−0.190 (0.292)	0.827 (0.466–1.467)	0.509
	Master’s Degree	−0.375 (0.334)	0.687 (0.359–1.336)	0.267
	Ph.D. or higher	−0.781 (0.370)	0.458 (0.222–0.945)	0.034 *
Protocol Status	No Protocol (Reference)	-	1.000	-
	Has Protocol	0.655 (0.270)	1.925 (1.134–3.268)	0.016 *

* Note: B, unstandardized regression coefficient; SE, standard error; OR, odds ratio; CI, confidence interval. Dependent Variable: Staff Safety Issue Experience (Yes = 1, No = 0). Significant at *p* < 0.05.

## Data Availability

The data presented in this study are available upon request from the corresponding author due to privacy and ethical reasons.

## Supplementary Materials

The following supporting information can be downloaded at: https://www.mdpi.com/article/10.3390/healthcare14131895/s1, File S1: English Translation of the Questionnaire. The original questionnaire was administered in Korean; an English-translated version is attached at the end of the main text as a supplementary file.

## Abbreviations

The following abbreviations are used in this manuscript:

CI	Confidence interval
OR	Odds ratio
KOPS	Korea Patient Safety Reporting & Learning
