# Supplementary material for: Patient and Staff Safety Incidents in Korean Dental Practice: Implications for Quality of Care and Safer Healthcare Delivery
Source: Healthcare (Basel). 2026 Jun 30;14(13):1895. doi: 10.3390/healthcare14131895 (PMC13362306; doi:10.3390/healthcare14131895)
Supplement: Supplementary file 1 [file healthcare-14-01895-s001.zip › healthcare-4372671-supplementary.pdf]

Supplementary File S1. English Translation of the Questionnaire

Questionnaire on Dental Patient Safety Incidents

This questionnaire was developed specifically for the present study and was originally administered in Korean. The following is the English translation used for supplementary documentation. This study was approved by the Institutional Review Board (IRB) of The Catholic University of Korea, Eunpyeong St. Mary's Hospital (Approval No. PC23QISI0037).

Participant Information and Voluntary Participation Statement

Hello. Thank you for your interest in this survey. This questionnaire is intended for research on factors affecting safety incidents in dental settings and on the prevention of dental safety incidents. The survey takes approximately 3-5 minutes to complete. Your responses will be used as valuable data to improve patient safety. All collected data are anonymous and confidential and will not be used for purposes other than this study. Participation is voluntary. You may stop participating at any time without any disadvantage.

Section I. General Characteristics

No.	Question (English translation)	Response options
1	What is your age?	- 20-29 years<br>- 30-39 years<br>- 40-49 years<br>- 50-59 years<br>- 60 years or older
2	What is your sex?	- Male<br>- Female
3	What is your occupation?	- Dentist / Physician<br>- Dental hygienist<br>- Nurse<br>- Other (specify)
4	What type of dental clinic or dental institution do you work in?	- Dental clinic<br>- Dental hospital<br>- Dental department in a general hospital<br>- Dental department in a tertiary general hospital (university hospital)<br>- Other (specify)
5	How often does your dental clinic treat patients with disabilities?	- Do not treat patients with disabilities<br>- Treat fewer than non-disabled (general) patients<br>- Treat a similar level as non-disabled (general) patients<br>- Treat more than non-disabled (general) patients<br>- Treat only patients with disabilities
6	What is your work department?	- Dental treatment room/operatory<br>- Dental front desk/reception<br>- Outpatient department outside dentistry<br>- Ward/inpatient unit<br>- Operating room<br>- Other (specify)
7	How many years of professional experience do you have?	- 1 year or less<br>- 2-3 years<br>- 4-5 years<br>- 6-10 years<br>- 11-20 years<br>- 21 years or more
8	What is your highest level of education?	- Associate degree<br>- Bachelor's degree<br>- Master's degree<br>- Doctoral degree<br>- Other (specify)

Section II. Experience of Dental Patient Safety Incidents

No.	Question (English translation)	Response options
9	Have you ever actually experienced a tooth- or dental-related safety incident involving a patient or healthcare staff member?	- Yes<br>- No
10	Please check all dental-related safety incidents you have experienced while working (multiple responses allowed). If not listed, please write it in.	- Swallowing of a tooth or prosthesis<br>- Fall accident<br>- Iatrogenic injury (e.g., tooth fracture/mobility/avulsion)<br>- Wrong-tooth treatment<br>- Injury to lips/oral cavity caused by dental instruments<br>- Infection-related problem<br>- Chemical injury to skin/clothing (e.g., NaOCl, etchant)<br>- Sensory nerve injury after dental treatment (e.g., implant surgery/extraction)<br>- Patient hyperventilation, syncope, cardiac arrest, etc.<br>- Allergy/hypersensitivity after injection/medication/treatment<br>- Puncture injury from instruments<br>- Laceration/cut from instruments<br>- Burn from chemicals or instruments<br>- Abrasion/scratch<br>- Bite injury caused by a patient<br>- Verbal abuse<br>- Physical assault<br>- Other (specify)
11	In the past 1 year, how many such dental safety incidents occurred in your institution?	- 0<br>- 1-2<br>- 3-5<br>- 6-10<br>- 11 or more
12	Do you know how to respond after a dental safety incident occurs?	- Yes<br>- No

Section III. Characteristics of Patients with Incidents and Response Measures

No.	Question (English translation)	Response options
13	In the past 1 year, please indicate all age groups of patients in whom you experienced a dental safety incident or near miss at your institution (multiple responses allowed). If none, select only No. 5.	- 1) Infants and preschool children<br>- 2) Children and adolescents (elementary, middle, and high school students)<br>- 3) Adults (19 years and older, under 65 years)<br>- 4) Older adults (65 years and older)<br>- 5) No dental safety incidents
14	How frequently do dental safety incidents (or near misses) occur among patients with disabilities?	- Do not treat patients with disabilities<br>- Less frequent than non-disabled (general) patients<br>- Similar frequency to non-disabled (general) patients<br>- More frequent than non-disabled (general) patients<br>- Much more frequent than non-disabled (general) patients
15	Does your dental clinic/hospital have a protocol for dental-related safety incidents?	- Yes<br>- No
16	Do you know preventive measures for safety incidents in your dental clinic?	- Yes<br>- No
17	Please indicate all devices or methods used in your dental clinic/hospital to prevent patient safety incidents (multiple responses allowed).	- Use of anti-swallowing devices/instruments<br>- Gauze packing, etc. to prevent swallowing<br>- Use of patient restraint/fixation devices (e.g., Papoose board)<br>- Use of rubber dam<br>- Fall-risk warning signage<br>- Identification and management of patients with infectious diseases<br>- Verification of treatment site through preoperative time-out<br>- Patient identification using two or more identifiers

		- Referral to a higher-level hospital for patients with systemic disease or disabilities<br>- Referral for dental evaluation before surgery/procedures (for physician/nurse cases)<br>- Other (specify)
18	Does your dental clinic or hospital have liability insurance?	- Yes<br>- No<br>- Not sure
19	Have you ever received help from liability insurance in relation to dental safety incidents?	- Yes<br>- No<br>- Not sure<br>- Not enrolled in liability insurance
20	Do you think liability insurance is helpful when a tooth/dental patient safety incident occurs?	- Very helpful<br>- Helpful<br>- Not sure<br>- Not helpful<br>- Not helpful at all<br>- Not enrolled in liability insurance

Section IV. Patient Safety Education

No.	Question (English translation)	Response options
21	Have you ever received education/training on prevention and response measures for tooth/dental-related safety incidents?	- Yes<br>- No
21-1	Where did you receive education/training on tooth/dental-related patient safety incidents?	- Mandatory education within clinic/hospital<br>- License continuing education<br>- Offline seminar/academic conference<br>- Online seminar/academic conference<br>- Other (specify)
21-2	Was the education/training actually helpful in your clinical practice?	- Very helpful<br>- Helpful<br>- Not sure<br>- Not helpful<br>- Not helpful at all
22	Do you think education/training on prevention and response measures for tooth/dental-related patient safety incidents is necessary?	- Very necessary<br>- Necessary<br>- Not sure<br>- Not necessary<br>- Not necessary at all
23	Do you think a shortage of clinical support staff or clinical personnel can be a cause of safety incidents?	- Very strongly related<br>- Related<br>- Neutral<br>- Not related<br>- Not related at all
24	When treating non-disabled (general) dental patients, what is the appropriate number of clinical staff (including dentists and dental hygienists)?	- 1 person<br>- 2 persons<br>- 3 persons<br>- 4 persons<br>- Other (specify)
25	Do you think additional clinical staff are needed when treating patients with disabilities?	- Very necessary<br>- Necessary<br>- Neutral<br>- Not necessary<br>- Not necessary at all
26	What is the appropriate number of clinical staff when treating patients with disabilities?	- 1 person<br>- 2 persons<br>- 3 persons<br>- 4 persons<br>- Do not know because I do not treat patients with disabilities
27	When treating patients with disabilities, how much more treatment time is required compared with non-disabled (general) patients?	- No difference<br>- 15 minutes or less more<br>- 16-30 minutes more<br>- 31-45 minutes more<br>- 46-60 minutes more

		- More than 1 hour more<br>- Do not treat patients with disabilities
28	Do you think the reimbursement fee for treatment of patients with disabilities is appropriate?	- Very low<br>- Low<br>- Neutral<br>- High<br>- Very high
29	Do you think increasing reimbursement fees would help promote dental treatment for patients with disabilities?	- Very helpful<br>- Helpful<br>- Neutral<br>- Not helpful<br>- Not helpful at all
30	If devices or materials are developed to ensure safety in patient care or in treating patients with disabilities, would you be willing to use them?	- Definitely would not use<br>- Would not use<br>- Neutral<br>- Would use<br>- Definitely would use
31	For anti-swallowing devices/materials, what is an appropriate price? (Assuming additional purchase is required)	- Less than KRW 1,000 (0.7 USD)<br>- Less than KRW 3,000 (2 USD)<br>- Less than KRW 5,000 (3 USD)<br>- Less than KRW 10,000 (6 USD)<br>- KRW 10,000 or more<br>- Would not use
32	Please provide any ideas or suggestions regarding dental treatment for patients with disabilities or prevention of dental-related safety incidents. (Optional free-text response)	- Open-ended response (optional)
